# Supplementary material for: Comparing standard and technology-assisted peer-delivered CBT for perinatal depression: A causal mediation study
Source: Glob Ment Health (Camb). 2025 Dec 9;13:e13. doi: 10.1017/gmh.2025.10109 (PMC12835944; doi:10.1017/gmh.2025.10109)
Supplement: Waqas et al. supplementary material [file S205442512510109Xsup001.docx]

**Supplementary Methods**

**Selection of mediators**

Mediators were included in the final model if they were associated with the exposure, another mediator, or the outcome whilst adjusting for mediator-outcome confounders (*p*<0.05). Using the above criteria, we can capture any potential mediator that is influenced by the intervention and influences either another mediator, or the improvement in depressive symptoms.

**Mediator-outcome confounders**

Due to the randomised nature of the exposure, it is not necessary to account for confounders of the association between the exposure and the outcome. However, it is necessary to account for baseline confounders (unaffected by the intervention) that were associated with the mediator and the outcome. These mediator-outcome confounders can generate spurious correlations between the mediator and outcome when unadjusted for, potentially distorting these associations. We considered 10 potential baseline characteristics that are not influenced by the intervention, as potential confounders. The selection process for these confounders is described in the section on *estimation methods* below.

**Decomposition of total effect of the CONEMO intervention into direct and indirect effects**

We decomposed the total effect of the ENHANCE intervention into interventional indirect effects via each of the five posited mediators and the direct effect via none of the mediators. The interventional indirect effect via a particular mediator (e.g., levels of perceived social support) can be interpreted as the average change in the potential outcome (improved symptoms of depression at six months) resulting from shifting the counterfactual distribution of that mediator from the exposed status (e.g., levels of perceived social support in the intervention arm) to the unexposed status (levels of perceived social support in the control arm), while setting each of the remaining mediators to random draws from either the exposed or unexposed group, depending on the specific decomposition. In doing so, valid inferences are not contingent on strict assumptions, such as correctly specifying the causal ordering among the mediators which are unlikely to be feasible in our current setting and can lead to incorrect inferences when violated. (1)

**Steps to estimation**

Estimation for the interventional indirect effects was based on Monte Carlo integration using a 1,000-fold expanded dataset. (15) The expanded dataset was created in four steps separately for each of the different sites.

In the first step, we fitted a model for each mediator given exposure and other predictors. Specifically, we fitted linear regression models for each of the six mediators to the observed data. Each model included a combination of predictors that were shown to be associated with the mediator of interest at the 5 % level including: baseline values of perceived levels of social support, baseline PHQ-9 scores, baseline anxiety levels captured using the GAD-7 instrument, baseline levels of disability captured using the WHO disability score, parity, nuclear family (yes/no), history of mental illness in the family (yes/no), cohabiting with partner, history of preterm deliveries, pregnancy duration, estimated household income, level of education. Interactions and non-linearities were explored and included if determined to be significant at the 5% level, using Stata’s post estimation command, *testparm*.

In the second step, the fitted mediator models were used to generate random, subject-specific Monte Carlo draws of each mediator for both the exposed and unexposed condition (i.e. counterfactual), given their observed covariate values.

In the third step, we fitted a model for the outcome, using a linear regression model for symptoms of depression captured using PHQ-9 scores, separately in the exposed and unexposed, given the mediators and mediator-outcome confounders. Any potential mediator-outcome confounder was included if it was associated with the mediator or the outcome (*p*<0.05). We used a model selection criterion similar to that of the mediator models; i.e., Any relevant non-linearities and interactions were included in the outcome model if determined to be significant at the five percent level, using the post-estimation *testparm* command in Stata.

Mediator-outcome confounders included in the analyses include baseline values of perceived levels of social support, baseline PHQ-9 scores, baseline anxiety levels captured using the GAD-7 instrument, baseline levels of disability captured using the WHO disability score, parity, nuclear family (yes/no), history of mental illness in the family (yes/no), cohabiting with partner, history of preterm deliveries, pregnancy duration, estimated household income, level of education.

Interactions between mediators and mediator outcome confounders were also examined for and included if the above-described criterion were fulfilled. Significant interactions were found between the following: levels of perceived social support (M3) and baseline symptoms of depression, levels of empathy (M5) and baseline symptoms of depression

In the fourth step, we used the fitted outcome model to predict the potential outcomes in the expanded dataset given the random, subject-specific draws of the mediator counterfactuals from the second step. The interventional indirect effects were then calculated as the average differences between potential outcomes under different hypothetical exposure levels.

**References**

1. Loh WW, Moerkerke B, Loeys T, et al. Heterogeneous indirect effects for multiple mediators using interventional effect models. Epidemiol Methods. 2020;9(1).

Supplementary table 1: **Baseline characteristics of participants in the ITT population**

| **Characteristic** | **THP-TAP (*n* = 487)** | **WHO-THP (*n* = 493)** |
| --- | --- | --- |
| Age (mean, s.d.) | 27.20 (5.15) | 27.29 (4.94) |
| Occupational status | | |
| Housewife | 473 (97.1%) | 479 (97.2) |
| Manual | 0 (0%) | 1 (0.2%) |
| Partially skilled | 2 (0.4%) | 6 (1.2%) |
| Professionals | 7 (1.4%) | 5 (1.0%) |
| Unskilled | 5 (1.0%) | 2 (0.4%) |
| Education | | |
| None | 46 (9.4%) | 41 (8.3%) |
| Primary | 67 (13.8%) | 41 (8.3%) |
| Middle-high | 244 (50.1%) | 275 (55.8%) |
| Intermediate | 73 (15.0%) | 89 (18.1%) |
| University | 57 (11.7%) | 47 (9.5%) |
| Number of children (mean, s.d.) | 2.03 (1.32) | 1.98 (1.32) |
| Previous miscarriages or stillbirth (mean, s.d.) | 1.61 (0.93) | 1.50 (0.94) |
| Occupational status of husband | | |
| Employed | 457 (93.8%) | 462 (93.7%) |
| Unemployed | 30 (6.2%) | 31 (6.3%) |
| PHQ-9 scores (mean, s.d.) | 16.73 (4.57) | 16.16 (4.51) |
| GAD-7 scores (mean, s.d.) | 11.79 (3.78) | 11.80 (4.09) |
| WHO-DAS scores (mean, s.d.) | 20.67 (8.58) | 20.10 (8.59) |

1. Data are number (%) or mean (s.d.).

Table adapted from Rahman et al., 2025

Reference: Rahman A, Malik A, Nazir H, Zaidi A, Nisar A, Waqas A, Atif N, Gibbs NK, Luo Y, Sikander S, Wang D. Technology-assisted cognitive-behavioral therapy for perinatal depression delivered by lived-experience peers: a cluster-randomized noninferiority trial. Nature Medicine. 2025 Apr 8:1-8.

Supplementary figure 1: CONSORT flow diagram


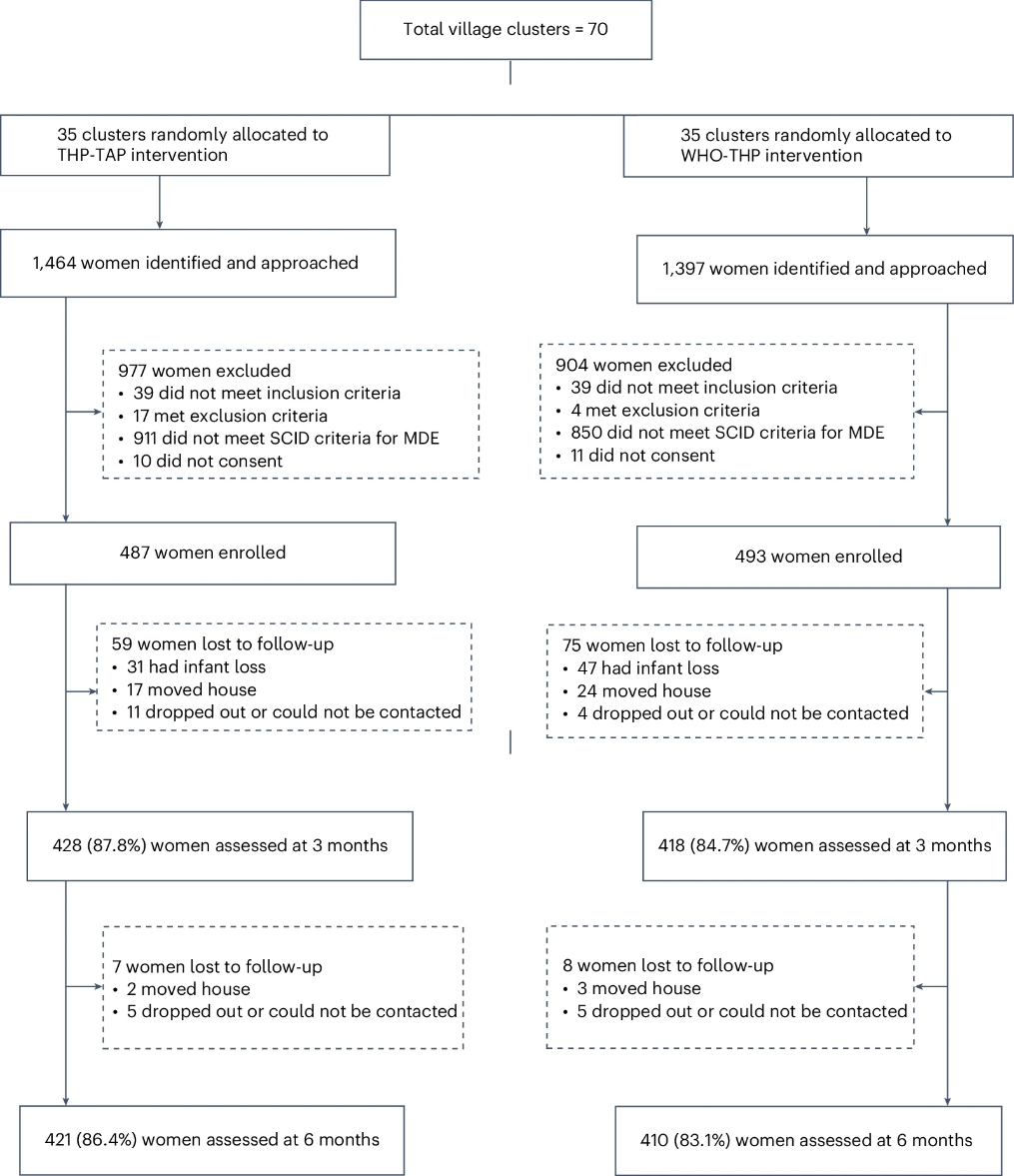


Figure adapted from Rahman et al., 2025; Reference: Rahman A, Malik A, Nazir H, Zaidi A, Nisar A, Waqas A, Atif N, Gibbs NK, Luo Y, Sikander S, Wang D. Technology-assisted cognitive-behavioral therapy for perinatal depression delivered by lived-experience peers: a cluster-randomized noninferiority trial. Nature Medicine. 2025 Apr 8:1-8.
